# Dupilumab-Induced Myalgia and Enthesitis in a Pediatric Patient Treated for Severe Atopic Dermatitis

**DOI:** 10.7759/cureus.96773

**Published:** 2025-11-13

**Authors:** Mohamad Sabsabee, Alaa Salman, Sheikha Alketbi, Mustafa Al Hamdani, Ali Nasreldien

**Affiliations:** 1 Pediatrics, Al Jalila Children's Speciality Hospital, Dubai, ARE; 2 Pediatric Neurology, Al Jalila Children's Speciality Hospital, Dubai, ARE; 3 Dermatology, Rashid Hospital, Dubai, ARE; 4 Pediatrics, Gulf Medical University, Dubai, ARE

**Keywords:** dupilumab, dupilumab adverse reaction, eczema, enthesitis, myalgia

## Abstract

Dupilumab is a monoclonal antibody systemic therapy approved for the treatment of atopic dermatitis (AD). It works by blocking the signaling pathways of interleukin-4 (IL-4) and interleukin-13 (IL-13). This case report describes a 10-year-old male with severe AD who developed myalgia and enthesitis suspected to be induced by dupilumab. The patient developed severe myalgia after a month of being on dupilumab, although his eczematous skin rash showed considerable improvement after treatment initiation. His pain was restricting movement and leading to contractures, which made him unable to walk or stand independently. A thorough diagnostic workup, which included rheumatological, brain, and spine imaging studies, was done to exclude other possible causes. Treatment with dupilumab was stopped, and the patient was advised to undergo physiotherapy and serial casting to restore his muscle function and for the correction of contractures.

While dupilumab is regarded as a safe and well-tolerated medication, this report illustrates uncommon adverse effects of the drug that are rarely described in the pediatric population and may contribute to considerable morbidity. A probable underlying mechanism may involve an interaction between the IL-4/IL-13 axis and the IL-17/IL-23 axis, which also play a role in autoimmune arthritis. The report focuses on the significance of close monitoring for musculoskeletal symptoms in patients receiving dupilumab and emphasizes the need for prompt drug discontinuation if such manifestations occur.

## Introduction

Atopic dermatitis (AD) is a common chronic inflammatory skin disorder characterized by the development of pruritus, dryness, and intermittent flares. It is a multifactorial disorder that characteristically develops in infancy or childhood and may continue into adulthood [1]. Worldwide, AD impacts millions of people, and conventional therapies have historically included topical corticosteroids, emollients, phototherapy, and calcineurin inhibitors.

Dupilumab, a fully human monoclonal antibody, is specifically designed to target the alpha subunit of the interleukin-4 receptor (IL-4Rα) [2]. These cytokines are central to the type 2 helper T-cell (Th2) immune response, which initiates inflammation, production of IgE, and recruitment of mast cells in atopic conditions like asthma, eosinophilic esophagitis, and AD. Clinical trials have demonstrated the strong efficacy of dupilumab, showing significant improvements in skin symptoms and overall quality of life [3].

Despite its overall favorable safety profile, dupilumab is associated with common adverse events such as injection site reactions, upper respiratory tract infections, and conjunctivitis. Of note, musculoskeletal complaints were not reported in the initial large-scale clinical trials. However, in recent years, a rising number of case reports have described the onset of dupilumab-induced arthropathy and enthesitis, encouraging a re-evaluation of its side effect profile [4]. This case report presents a rare occurrence of myalgia and enthesitis in a pediatric patient receiving dupilumab for severe AD.

Consent for publication of this case report and any accompanying images was obtained from the patient’s parent.

## Case presentation

Initial presentation

**Figure 1:**
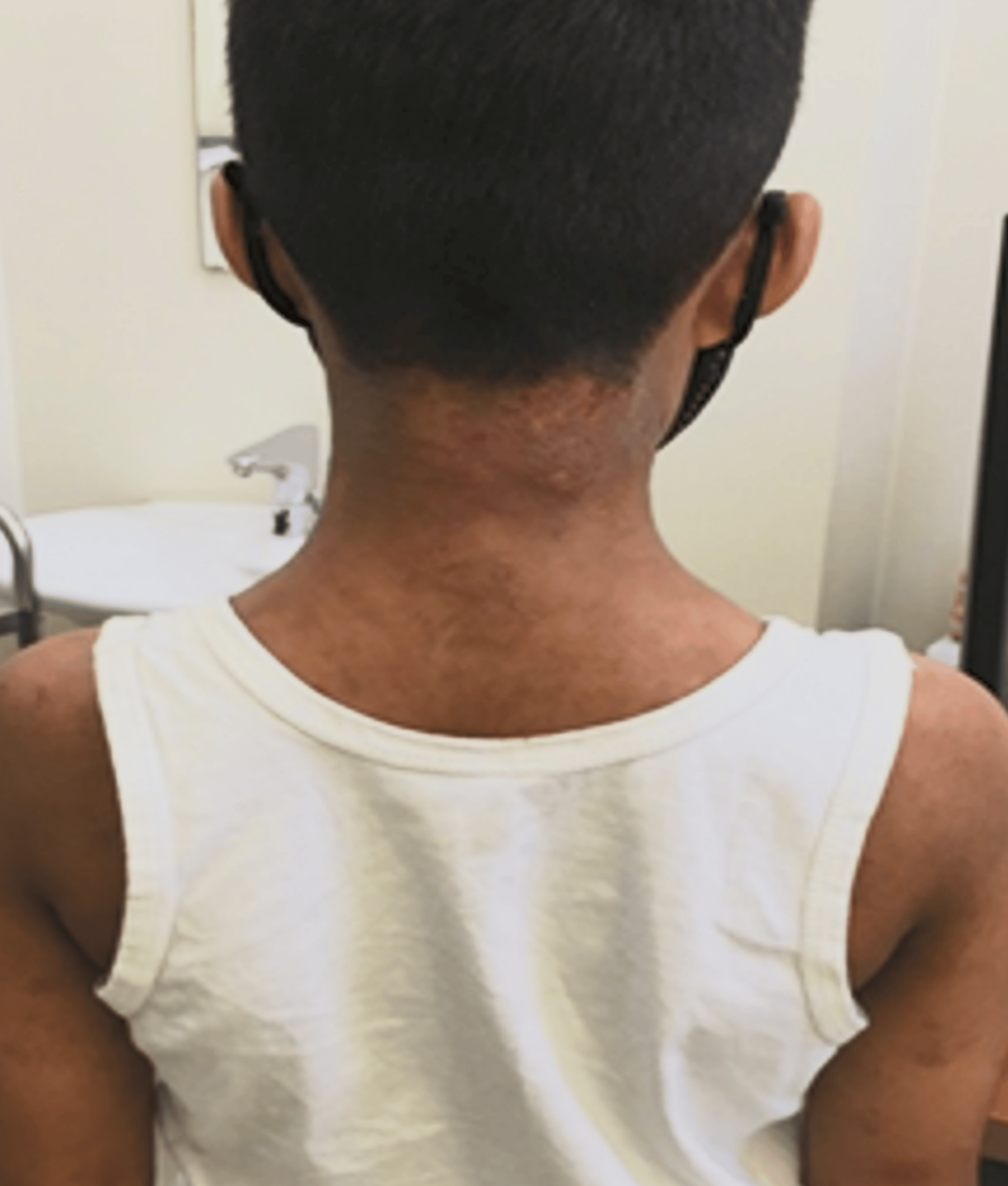
Atopic dermatitis on the neck (before dupilumab treatment)

The patient was a 10-year-old male with a three-year history of severe AD (Figure 1). He was initially started on cyclosporine 50 mg twice daily, which was gradually tapered and discontinued after two months due to adverse effects, including aggressive behavior, agitation, and anxiety, as well as hypertension that was unresponsive to medication. Hence, dupilumab injections were initiated at a loading dose of 600 mg, followed by 300 mg every two weeks. After four weeks, when the fourth dose was due, his eczematous rash had improved. Nonetheless, he continued to experience severe pruritus, along with agitation, aggressive behavior, arthralgia, and myalgia. He was therefore admitted for further evaluation and management.

Hospital course

**Figure 2:**
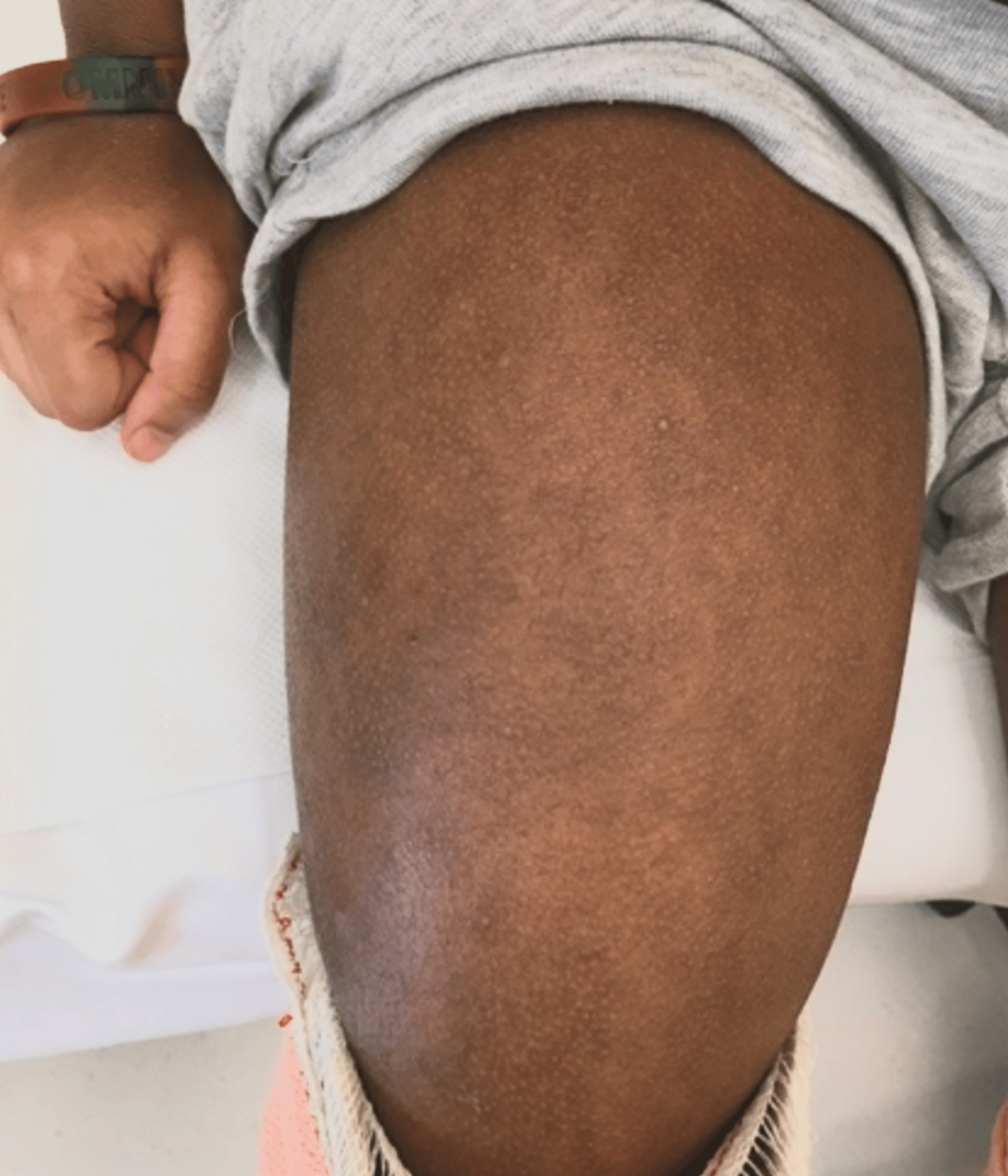
Skin of the thigh, the primary location of myalgia and arthralgia

During hospital admission, the pruritus and pain were so severe that he refused to wear clothes or be touched by anyone. He was kept on antihistamines, skin emollients, and IV midazolam for sedation. On another note, he complained of increasing thigh pain (over the area seen in Figure 2). The pain prevented him from standing or getting up to use the bathroom. Pelvic MRI findings were unremarkable, and bilateral knee extension casts were applied due to bilateral contractures. Investigations for rheumatological disorders were negative (Table 1). A skin biopsy was performed and showed psoriasiform dermatitis with superficial and deep perivascular Inflammation. Hyper IgE syndrome was initially suspected; however, immunoglobulin levels were within normal limits, and genetic testing was negative. He was assessed by a psychiatry team who advised psychotherapy alone with “as needed” anxiolytics. Brain MRI was unremarkable apart from nonspecific T2/FLAIR hyperintensity foci in the white matter. At the time of discharge, his symptoms had improved, and lower limb strength was normal. Dupilumab was discontinued, and he was discharged with bilateral removable casts and topical treatments for eczema.

**Table 1:** Lab results WBC: white blood cell; RBC: red blood cell; CPK: creatine phosphokinase; LDH: lactic dehydrogenase; IgE: immunoglobulin E; ANA: antinuclear antibody

Variable	Patient value	Normal range
WBC count, x10^3^/uL	14.2	4.5 - 13.5
RBC count, x10^3^/uL	4.62	4.0 - 5.2
Hemoglobin, g/dL	12.2	11.5 - 15.5
Hematocrit, %	39.10	35 - 45
Platelet count, x10^3^/uL	417	150 - 450
Neutrophils absolute, x10^3^/uL	4.54	1.5 - 8.5
Lymphocytes absolute, x10^3^/uL	6.11	1.5 - 7.0
Monocytes absolute, x10^3^/uL	0.57	0.2 - 1.0
Eosinophils absolute, x10^3^/uL	2.84	0.0 - 0.5
Basophils absolute, x10^3^/uL	0.14	0.0 - 0.2
Uric acid, mg/dL	4.1	2.0 - 5.5
CPK, U/L	136	60 - 305
LDH, U/L	481	180 - 430
IgE, IU/ml	16,320	<100
IgG, g/L	13.34	7.0 - 16.0
IgA, g/L	2.46	0.4 - 2.5
IgM, g/L	0.74	0.4 - 2.3
ANA panel	Negative	Negative
Hyper IgE syndrome genetic panel	Negative	Negative

Follow-up

The patient continued following up with the mental health team, who assessed him again and decided to start fluoxetine 5 mg OD given his persistent depressed mood and impaired concentration; at a later follow-up, his mother noted improvement with the medication. He was not compliant with physiotherapy and casting due to pain. Four months later, he was seen in the allergy clinic as his eczema had flared up again. At this point, he was brought in a wheelchair. While his myalgia and arthralgia had resolved, residual effects remained. Bilateral contractures of elbows, knees, and ankles were present and more prominent on the left side. He could not stand without support and could not walk properly. Serial CPK measurements were within normal limits.

**Figure 3:**
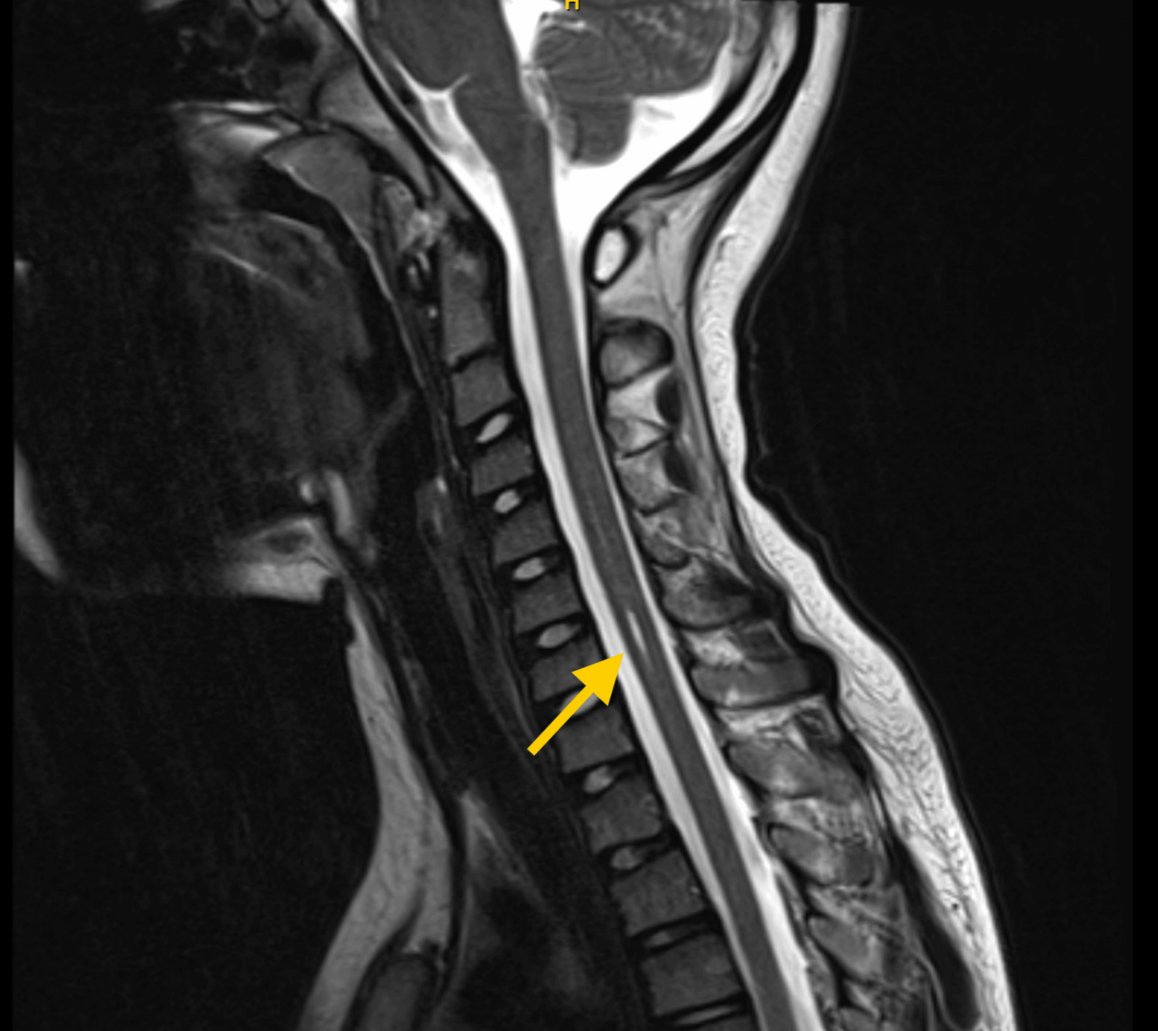
MRI T2-weighted image of cervical syringomyelia (arrow) MRI: magnetic resonance imaging

The management was overseen by a multidisciplinary team. For contractures, the patient underwent serial casting with release procedures. Even though he regained the ability to walk independently, increased lower limb tone with exaggerated reflexes was noted. He underwent an MRI of the brain and spine, which revealed a tiny syringomyelia in the cervical cord (Figure 3), which did not account for the lower limb findings and did not require intervention according to the neurologist and neuroradiologist.

On subsequent follow-ups, the patient had returned to normal activities, including walking, running, and jumping. His myalgia had resolved; however, his eczema continued to flare, suggesting dupilumab as the underlying cause.

## Discussion

The bilateral thigh pain experienced by our patient started shortly after initiating dupilumab. A thorough diagnostic workup, including brain and spine imaging and extensive laboratory testing, failed to identify any cause other than a potential adverse reaction to the medication. The tiny cervical syrinx, an incidental finding on imaging, was unlikely to explain his lower limb pain, as a syrinx of this size typically would not produce such symptoms. Neurological deficits from a cervical syrinx would also be expected to appear in the upper limbs before affecting the lower limbs. Primary differential diagnoses encompassed a possible rheumatological disorder. However, this was deemed unlikely given a negative autoimmune workup, lack of arthropathy, and a negative family history. Consistently normal CPK levels further excluded a classic myopathy.

An interesting cohort study by Ariëns et al. involving 210 adult patients with AD managed with dupilumab reported a 7.6% incidence of muscle and/or joint pain [5]. Similarly, a review by Jay et al. on dupilumab-associated inflammatory arthritis found that symptoms typically develop within four months of starting the medication, although onset can occur much later. The authors noted that symptom severity at onset strongly predicts the clinical course: while mild cases can be managed with nonsteroidal anti-inflammatory drugs (NSAIDs) without stopping dupilumab, more severe cases require immediate discontinuation of the medication. Our patient’s case, however, was more severe, with myalgia significant enough to impair mobility and cause contractures, necessitating more aggressive interventions [6].

The exact mechanism behind dupilumab-associated musculoskeletal side effects is still being explored. A study by Harb and Chatila explained that dupilumab works by blocking the shared IL-4Rα chain, thereby preventing signaling from both IL-4 and IL-13, which are key drivers of allergic ailment. However, a proposed explanation for the resulting arthropathy is that this blockade may modify the immune balance, potentially tilting it toward the IL-17/IL-23 inflammatory axis, which is recognized to be involved in autoimmune arthritis [7].

A recent review by Astry et al. highlighted the IL-23/IL-17 axis as a key pathogenic driver, with Th17 cells playing a major role in immune-mediated pathology. It is hypothesized that dupilumab inhibition of IL-4 may interrupt the immune equilibrium, leading to a compensatory upregulation of the IL-17/IL-23 pathway [8]. A review by Bridgewood et al. provides a critical immunological context, clarifying that the IL-23/IL-17 axis, which drives spondyloarthropathies, and the IL-4/IL-13 axis, which drives allergic ailments, are not mutually exclusive [9]. They describe that a Th2 cytokine-driven immune response acts as an "immunological brake" on the IL-17 path. Thus, when this brake is removed by drugs like dupilumab, a spondyloarthropathy-like phenotype may appear in susceptible individuals.

## Conclusions

While dupilumab is normally a harmless drug with a favorable safety profile, it can, in rare instances, cause serious musculoskeletal adverse effects such as myopathy and enthesitis. Prompt recognition and drug discontinuation can prevent long-term morbidity. Further investigations are needed to thoroughly comprehend the mechanism of dupilumab-induced myopathy and to identify predisposing factors.
